# Megakaryocytes promote bone formation through coupling osteogenesis with angiogenesis by secreting TGF-β1

**DOI:** 10.7150/thno.40559

**Published:** 2020-01-12

**Authors:** Yong Tang, Mengjia Hu, Yang Xu, Fang Chen, Shilei Chen, Mo Chen, Yan Qi, Mingqiang Shen, Cheng Wang, Yukai Lu, Zihao Zhang, Hao Zeng, Yong Quan, Fengchao Wang, Yongping Su, Dongfeng Zeng, Song Wang, Junping Wang

**Affiliations:** State Key Laboratory of Trauma, Burns and Combined Injury, Institute of Combined Injury, Chongqing Engineering Research Center for Nanomedicine, College of Preventive Medicine, Third Military Medical University, Chongqing 400038, China.

**Keywords:** megakaryocyte, bone formation, angiogenesis, irradiation, TGF-β1

## Abstract

**Rationale**: The hematopoietic system and skeletal system have a close relationship, and megakaryocytes (MKs) may be involved in maintaining bone homeostasis. However, the exact role and underlying mechanism of MKs in bone formation during steady-state and stress conditions are still unclear.

**Methods**: We first evaluated the bone phenotype with MKs deficiency in bone marrow by using c-Mpl-deficient mice and MKs-conditionally deleted mice. Then, osteoblasts (OBs) proliferation and differentiation and CD31^hi^Emcn^hi^ tube formation were assessed. The expression of growth factors related to bone formation in MKs was detected by RNA-sequencing and enzyme-linked immunosorbent assays (ELISAs). Mice with specific depletion of TGF-β1 in MKs were used to further verify the effect of MKs on osteogenesis and angiogenesis. Finally, MKs treatment of irradiation-induced bone injury was tested in a mouse model.

**Results**: We found that MKs deficiency significantly impaired bone formation. Further investigations revealed that MKs could promote OBs proliferation and differentiation, as well as CD31^hi^Emcn^hi^ vessels formation, by secreting high levels of TGF-β1. Consistent with these findings, mice with specific depletion of TGF-β1 in MKs displayed significantly decreased bone mass and strength. Importantly, treatment with MKs or thrombopoietin (TPO) substantially attenuated radioactive bone injury in mice by directly or indirectly increasing the level of TGF-β1 in bone marrow. MKs-derived TGF-β1 was also involved in suppressing apoptosis and promoting DNA damage repair in OBs after irradiation exposure.

**Conclusions**: Our findings demonstrate that MKs contribute to bone formation through coupling osteogenesis with angiogenesis by secreting TGF-β1, which may offer a potential therapeutic strategy for the treatment of irradiation-induced osteoporosis.

## Introduction

Bone is a specific organ that is maintained by the balance of osteoblasts (OBs) and osteoclasts (OCs). During bone remodeling, OC-induced bone resorption and OB-induced bone formation promote the migration and differentiation of their precursors through endocrine and paracrine routes 1. An adequate blood supply can transport the nutrients necessary for the proliferation and differentiation of OBs, which is critical for bone homeostasis 2, 3. Therefore, an effective combination of angiogenesis and bone formation is essential for the bone metabolic balance. There are two subtypes of vascular endothelial cells (ECs): the H-type (referred to CD31^hi^Emcn^hi^ vessels) and the L-type (CD31^lo^Emcn^lo^ vessels). Osteoprogenitor cells prefer to be in contact with H-type ECs, because they are enriched in growth factors that are needed for OBs survival and proliferation 4, 5. However, the underlying mechanism by which H-type ECs couple osteogenesis and angiogenesis is unclear.

Bone damage induced by irradiation is a common side effect of radiotherapy and often leads to pathological fractures and other complications 6-8. The mechanism of the impaired bone formation induced by irradiation is very complex and involves cell cycle arrest, decreased differentiation of OBs and increased apoptosis of OBs 9-11. Moreover, irradiation can also reduce vascular ECs and subsequently impede the blood supply to bones, aggravating the bone injury 12-15. Nevertheless, the exact mechanism of irradiation-induced osteoporosis is unknown. Currently, bisphosphonates and parathyroid hormones, which can inhibit bone resorption and promote bone formation, respectively, are commonly used for the treatment of irradiation-induced osteoporosis. However, the long-term effect is unclear, and the clinical outcomes are not satisfactory 16-19. Therefore, identification of new targets to promote bone formation in patients subjected to tumor radiotherapy is urgently needed.

The hematopoietic system and skeletal system have a close relationship. OBs can affect the homeostasis of hematopoietic stem cells, as well as the generation of megakaryocytes (MKs) and platelets 20-23. Conversely, MKs can modulate the bone metabolic balance by secreting various growth factors 24-26. As shown in previous studies, mice lacking GATA-1 or NF-E2 displayed a substantial increase in MKs, accompanied by an increase in bone trabecular number and cortical bone thickness 27, 28. In addition, overexpression of thrombopoietin (TPO) or continuous injection of TPO in mice can result in high levels of MKs, which eventually lead to osteosclerosis 29, 30. Surprisingly, c-Mpl^-/-^ mice (the number of MKs was decreased by about 80%) displayed increased number of trabeculae with aging, while cortical bone thickness and strength were decreased 31. However, how MKs regulate bone formation during steady-state conditions and after irradiation is still unclear.

Here, we demonstrated that MKs can couple osteogenesis with angiogenesis, thereby regulating bone homeostasis. Further, our data exhibit the therapeutic effect of MKs on impaired OBs after irradiation through secretion of TGF-β1, and provide a new avenue to treat osteoporosis in patients undergoing radiotherapy.

## Materials and Methods

### Animals

C57BL/6J-Mpl^hlb219^/J mice, C57BL/6-Tg (Pf4-cre) Q3Rsko/J mice and C57BL/6-Gt (ROSA)26Sor^tm1(HBEGF)Awai^/J (iDTR) mice were obtained from the Jackson Laboratory. Pf4-cre^+^; iDTR mice were injected with vehicle or DT (at the dose of 50 ng/g body weight) every two days. Two weeks after first injection, these mice were used for subsequent analysis. Tgfb1tm2.1Doe/J (TGF-β1^fl/fl^) mice were purchased from Biocytogen Co.,Ltd (Beijing, China). For dynamic histomorphometric analysis, mice were separately injected with calcein (10 mg/kg) 10 and 3 days before sacrifice. Total body irradiation (TBI) of mice was performed as we previously described 32. All mice were treated following the guidelines of the committee on animal care (Third Military Medical University).

### Preparation of MKs, OBs and ECs

Primary MKs, OBs, and ECs were isolated according to previously published methods^21^, ^31^-^34^. For MKs preparation, c-kit^+^ cells from mouse bone marrow (BM) were first sorted with flow cytometry. Then, the cells were grown in StemSpan SFEM medium (Stem Cell Technologies, Vancouver, BC, Canada) in the presence of 20 ng/mL rmTPO (Peprotech; Rocky Hill, NJ, USA) and 10 ng/mL IL-3 (Peprotech). After 9-10 days of culture, the primary MKs were purified with flow cytometry according to the expression of CD41 (MWREG30; Biolegend, San Diego, CA, USA) and CD42b (M040-1; Emfret Analytics, Eibelstadt, Germany). For OBs preparation, calvaria obtained from neonatal mice was sequentially digested with 200 U/mL collagenase (Roche, Indianapolis, USA). The samples were incubated at 37°C for five different time periods (0-10, 10-20, 20-35, 35-50 and 50-65 min), and fractions 3-5 were collected and used for the OBs culture. ECs isolated from the BM of 7- to 8-week-old C57BL/6 mice were purchased from BioChain (Z7030031; Newark, CA, USA). These cells were positive for CD31 (MEC13.3; Santa Cruz, Dallas, Texas, USA), CD105 (MJ7/18; Biolegend) and vascular endothelial growth factor receptor 1 (VEGF-R1; ab9540, Abcam), and negative for CD133 (315-2C11; Biolegend). All experiments were performed on fresh, low passage (p2-3) cells.

### Coculture assays

For coculture experiments, OBs were plated onto 6-well plates (20×10^3^ cells/well). Twenty-four hours later, MKs (20×10^3^ cells/well) were plated into wells for direct coculture. For indirect coculture, OBs were seeded in the upper chamber of a 0.4 μm transwell insert (Corning, New York, USA) and MKs were seeded in the lower wells. Specified cultures were pretreated with 100 nM of SB431542 (Sigma-Aldrich, St. Louis, Missouri, USA) for OBs for approximately 1 h. MKs conditioned medium (MKs-CM) was obtained by removing MKs via centrifugation (5000 rpm, 10 min). In some assays, neutralizing antibodies against TGF-β1 (9016; R&D, Minneapolis, Minnesota, USA ), VEGF (AF-493; R&D), BMP6 (MAB6325; R&D), IGF-1 (AF791; R&D), PDGF-BB (ab34074; Abcam), CXCl12 (79014; R&D), BMP2 (MAB111; R&D), TGF-β2 (AB-112-NA; R&D), TGF-β3 (20724; R&D), BMP4 (MAB50201; R&D) and IgG (AB-108-C; R&D) were added to the MKs-CM.

### Tube formation

Matrigel matrix (Corning) was added to 24-well culture plates (289 μL per well) on ice and then incubated at 37°C for 60 min. ECs (12×10^4^ cells in 300μL per well) or combined OBs (6×10^4^ cells in 150μL per well) and ECs (12×10^4^ cells in 150 μL per well) were seeded on polymerized Matrigel with MKs-CM. After incubation on the angiogenesis assay plate for 4 h at 37 °C, tube formation was observed with a microscope (Olympus, Tokyo, Japan) and cumulative tube length was measured.

### Transplantation assays

Irradiated (9 Gy) WT recipient mice received 5×10^7^ spleen cells from WT or c-Mpl^-/-^ donor mice via tail vein injection. The femurs of these recipients were collected 4 weeks after transplantation.

### Establishment of the radioactive bone injury model

Mice were subjected to 6.5 Gy and 3.5 Gy irradiation at day 1 and day 14, respectively. After the first irradiation, the mice were intraperitoneally treated with vehicle or TPO every other day, or locally treated with vehicle or MKs (5×10^6^ in 10 μL) every week 35. For local treatment, the mice were anesthetized, and the distal femurs were gently drilled with a 26-gauge microsyringe through the patellar tendon. Then, vehicle or MKs were injected into the bone cavity through the hole in the femur. Two weeks or two months after continuous TPO or MKs treatments, the mice were used for subsequent analyses.

### Flow cytometric analysis

BM MKs were stained with anti-mouse CD41, CD45 (30-F11; Biolegend) and CD61 (154-2C11; Biolegend) antibodies. BM ECs were stained with anti-mouse CD31 and endomucin (V.7C7; Santa Cruz) antibodies. The cell cycle and apoptosis were analyzed using a BD Biosciences system (San Jose, CA, USA) according to the kit instructions. After direct or indirect coculture, the growth of CD41^-^ CD45^-^ OBs was assessed as described previously 36. Flow cytometric analysis was performed on a FACS-Verse system (BD Biosciences), and data were analyzed using FlowJo 10.3.0 (Tree Star, USA).

### Enzyme-linked immunosorbent assays (ELISAs)

The expression levels of growth factors in BM were detected as previously described 36. The kits used for measuring TGF-β1, VEGF, BMP6, IGF-1, PDGF-BB, CXCl12, BMP2, TGF-β2, TGF-β3 and BMP4 were purchased from R&D Systems.

### Microcomputed tomography (micro-CT)

Micro-computed tomography (Micro-CT Skyscan 1272 system; Bruker, Belgium) with an isotropic voxel size of 10 µm was used to quantify the bone parameters of the femurs as previously described 37. The scanning voltage was 60 kV, the current was 165 µA and the resolution was 10 µm per pixel. For trabecular bone analysis of the distal femur, starting from 10 layers after the distal growth plate disappeared, we selected 100 layers (1 mm) from the proximal femur as the trabecular bone and reconstruction area. For cortical bone analysis of the femur, 100 layers (1 mm) were taken from the midpoint of the femur to each end as the bone cortex area for analysis and reconstruction. Trabecular and cortical bones were thresholded at 90-255 (8 bit grayscale bitmap). Reconstruction was performed by Nrecon (Ver. 1.6.10). Three-dimensional (3D) images were obtained from contoured 2D images by methods based on distance transformation of the grayscale original images (CTvox, Ver. 3.0.0). The bone parameters, including bone mineral density (BMD, g/cm^3^), trabecular bone volume fraction (BV/TV, %), trabecular number (Tb.N, 1/mm), trabecular thickness (Tb.Th, mm), trabecular separation (Tb.Sp, mm) and cortical thickness (Ct. Th, mm), were calculated by CT Analyzer (Version 1.15.4.0, Belgium).

### Bone histomorphometry

The femurs were fixed, dehydrated, embedded without decalcification and sliced as previously described 38. Undecalcified femoral sections were stained with the von-Kossa method or left unstained to calculate the dynamic morphometric parameters. The mineral apposition rate (MAR)and bone formation rate per bone surface (BFR/BS) were analyzed with laser confocal microscope (LSM780; Carl Zeiss, Oberkochen, Germany). Decalcified femoral sections underwent HE and Masson staining for analysis of static parameters. Osteoblast surface (the percentage of trabecular bone surface covered by osteoblasts, Ob.S/BS) and osteoblast number/bone perimeter (Ob.N/B.Pm) were analyzed by Image J software (Image J, NIH, USA).

### Biomechanics

The relative bone strength was determined by a three-point bending test with a mechanical analysis instrument (Dynacell Life Sciences, LLC, Spring House, PA, USA) as previously described 39, 40. In brief, the femur was placed on two parallel scaffolds at a horizontal distance of 6 mm. Then, the load was applied vertically downward at a speed of 0.05 mm/s in the middle of the femur until it was broken. The load of peak (N) and stiffness (N/mm) were recorded.

### Angiography

Micro-CT was used to image bone vessels as previously described 41, 42. In brief, the mice were euthanized, the thoracic cavity was opened, and a needle was inserted into the left ventricle to establish an inflow tract. An outflow tract was established in the right auricle. Next, heparinized saline (5 mL, 100 U/mL), 10% neutral formalin (3 mL) and silicone rubber compound (3 mL, Microfil MV-122, Flow Tech, Massachusetts, USA) were injected. The specimens were stored at 4°C overnight and then fixed, and decalcified for approximately 21 days. Images were scanned by micro-CT, and the vascular volume and vascular surface area were calculated.

### Immunostaining

Immunofluorescence was performed as previously described 43, 44. Briefly, the bone sections were incubated with individual primary antibodies against mouse CD31 (ab28364; Abcam), endomucin (V.7C7; Santa Cruz), Ki67 (AF7617; R&D) and H2AX-Phospho (Ser139) (2F3; BioLegend) overnight at 4°C. Subsequently, the samples were incubated with fluorescent-coupled secondary antibodies for 1 h at 37°C in the dark. Images were collected using a confocal laser microscope (LSM780; Carl Zeiss).

Immunohistochemistry was performed with primary antibodies against osteocalcin (ab13420; Abcam), type I collagen (ab34710; Abcam) and cleaved caspase-3 (5A1E; Cell Signaling Technology, Danvers, MA, USA) at 4°C overnight. Then, the bone sections were incubated with biotinylated secondary antibody, avidin-biotin enzyme reagents, and the chromogen 3,3'-diaminobenzidine (DAB) tetrahydrochloride. Apoptosis of OBs was analyzed by TUNEL staining using an in situ cell death assay (Roche). The number of positive cells was normalized by Image J software (Image J, NIH, USA).

### Detection differentiation of OBs

OBs were seeded in a 12-well plate (50×10^3^ cells/well). When the cells reached 90% confluence, they were incubated in differentiation induction medium. After 7 days of culture, the alkaline phosphatase in these cells was stained using an alkaline phosphatase assay kit (Beyotime, Haimen, China) or detected with an alkaline phosphatase activity kit (Roche). After 3 weeks of culture, Alizarin Red was used for staining or quantitative analysis of mineralization (Sigma-Aldrich). Protein concentrations were determined using the BCA method to standardize the data (Beyotime).

### Real-time quantitative PCR (qPCR)

Total RNA was isolated using TRIzol reagent (Invitrogen, Carlsbad, CA, USA). Then, RNA was used to produce complementary DNA (cDNA) using the PrimeScript RT-PCR kit (DRR014A) (TaKaRa, Tokyo, Japan). Next, qPCR was performed using SYBR Premix Ex Taq II (TaKaRa) according to the manufacturer's instructions. The primer sequences are listed in Table S1.

### Western blotting

OBs or ECs were lysed using ice-cold lysis buffer containing 1% protease inhibitors and phosphatase inhibitors (Roche Applied Science, Penzberg, Germany). Proteins were fractionated by sodium dodecyl sulfate (SDS)-polyacrylamide gel electrophoresis and then transferred to polyvinylidene difluoride (PVDF) membranes. Protein expression was detected by incubation with anti-phospho-Smad2/3 (D27F4; Cell Signaling Technology), HIF-1α (D1S7W; Cell Signaling Technology), VEGF (ab52917; Abcam), or β-actin (AF0003; Beyotime) antibodies.

### RNA-sequencing (RNA-seq)

Human primary MKs were induced and cultured as previously reported 32. MKs from 14 d cultures were collected, and RNA was isolated. cDNA library construction and RNA-seq were performed at Genergy Bio (Shanghai, China). RNA-seq was performed using an Illumina HiSeq2000 system. The expression of known genes was assessed by fragments per kilobase of transcript per million fragments mapped (FPKM). All raw data were deposited in the National Center for Biotechnology Information (NCBI) database (PRJNA561251).

### Statistical analysis

The results of at least 3 independent experiments are expressed as the means ±SD. Differences between two groups and multiple groups were compared by two-tailed Student's t test and one-way analysis of variance (ANOVA), respectively. P<0.05 was considered statistically significant.

## Results

### MKs deficiency in BM impairs bone formation

**Figure 1:**
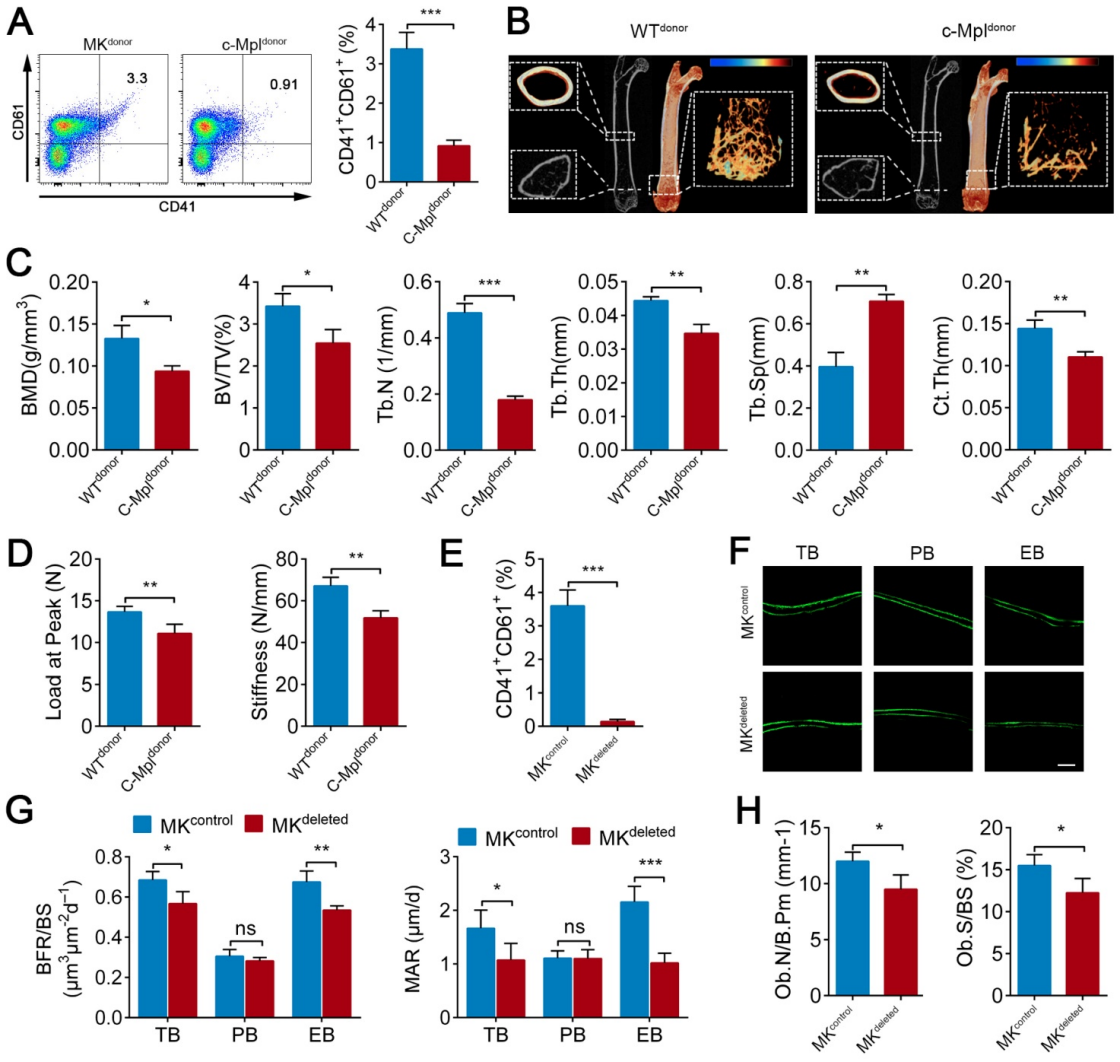
** MKs deficiency in BM impair bone formation.** (**A**) Flow cytometric analysis of the percentage of CD41^+^ CD61^+^ MKs in the BM of the mice reconstituted with c-Mpl^-/-^ or WT spleen cells (n=6 mice per group). Representative flow cytometry plots are shown in the left panel. (**B**) Representative Micro-CT images of longitudinal section femurs, cross-sectional view of the distal femurs and reconstructed trabecular structure of the ROI (White dashed box) from mice reconstituted with c-Mpl^-/-^ or WT spleen cells (n=6 mice per group). Color scale bar represents bone mineral density level. (**C**) Quantitative Micro-CT analysis of the bone mineral density (BMD), trabecular bone fraction (BV/TV, Tb.N, Tb.Th and Tb.Sp) and cortical thickness (Ct.Th) of femora from mice reconstituted with c-Mpl^-/-^ or WT spleen cells (n=6 mice per group). (**D**) Quantitative biomechanical analysis of femora (Load of peak and stiffness) from mice reconstituted with c-Mpl^-/-^ or WT spleen cells (n=6 mice per group). (**E**) Flow cytometric analysis of the percentage of CD41^+^ CD61^+^ MKs in the BM of MK^deleted^ mice and their littermate controls (n=6 mice per group).(**F**) Representative images of calcein double labeling of trabecular (TB), endocortical (EB) and periosteal bone (PB) from MK^deleted^ mice and their littermate controls (n=6 mice per group). Scale bar, 20 µm. (**G**) Quantification of mineral apposition rate (MAR) and bone formation rate (BFR) for MK^deleted^ mice and their littermate controls (n=6 mice per group). (**H**) The bone histomorphometry parameters (Ob.S/BS, Ob.N/B.Pm) at the distal femur metaphysis from MK^deleted^ mice and their littermate controls (n=6 mice per group). Data are shown as mean ± SD. *P < 0.05, **P < 0.01, ***P < 0.001. For all panels in this figure, data are representative of three independent experiments.

Previous studies, including a report from our group, have found that c-Mpl knockout leads to a notable decrease in MKs number in mice 45. Furthermore, five-month old c-Mpl^-/-^ mice showed decreased cortical bone thickness and bone strength 31. To investigate the role of MKs in bone homeostasis, we generated a mouse model with MKs deficiency by transferring c-Mpl^-/-^ spleen cells into lethally irradiated recipient mice (WT). The mice reconstituted with c-Mpl^-/-^ spleen cells displayed an approximately 73% decrease in MKs compared to the control mice (Figure 1A). Notably, these recipient mice developed a bone loss phenotype 4 weeks after transplantation (Figure 1B), accompanied by significant differences in bone parameters, including BMD, BV/TV, Tb.N, Tb.Th, Tb.Sp and Ct.Th (Figure 1C). Furthermore, biomechanical analysis showed that the bone strength and stiffness declined by approximately 18.8% and 22.8%, respectively, in mice reconstituted with c-Mpl^-/-^ spleen cells (Figure 1D).

To assess whether MKs affect osteogenesis, we introduced a Pf4-cre mice that is widely used in MK-associated investigations despite a low-level ectopic recombination of Pf4 (Figure S1A-C). Then, inducible MK-deleted mice (Pf4-cre; iDTR) were generated by crossing Pf4-cre mice with iDTR mice 46. Flow cytometric analysis revealed that the CD41^+^CD61^+^ MKs were decreased by approximately 96% in the BM of Pf4-cre^+^; iDTR (hereafter referred to as MK^deleted^) mice after diphtheria toxin (DT) injections (Figure 1E; Figure S1D). Importantly, undecalcified bone histological analysis showed that both the MAR and BFR/BS were decreased in the MK^deleted^ mice compared with the littermate controls (Figure 1F, G). Moreover, bone histomorphometry analysis showed that the Ob.S/BS and Ob.N/B.Pm were significantly lower in the MK^deleted^ mice than their littermate controls (Figure 1H). Taken together, these data suggest that MKs are involved in bone formation.

### MKs promote OBs proliferation and differentiation, as well as ECs tube formation

**Figure 2:**
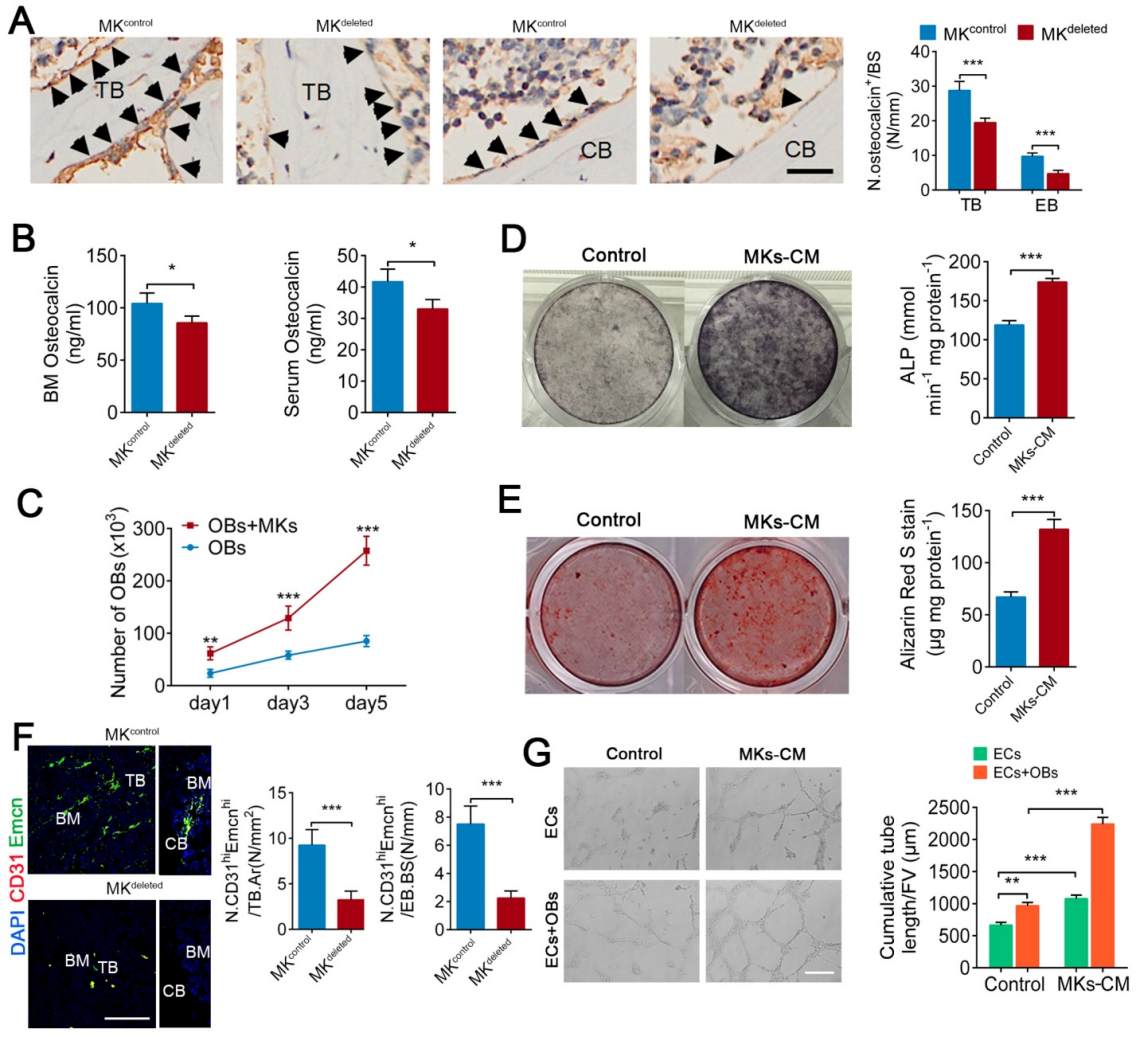
** MKs promote OBs proliferation and differentiation, as well as ECs tube formation.** (**A**) Representative immunostaining images of osteocalcin^+^ cells (arrowheads) on the surfaces of TB and EB from MK^deleted^ mice and their littermate controls (n=6 mice per group). The quantification of osteocalcin^+^ cells are shown in the right panel. Scale bar, 100 µm. (**B**) The concentration of osteocalcin in the BM and serum of MK^deleted^ mice and their littermate controls, determined by ELISA (n=6 mice per group). (**C**) Proliferation of OBs in direct culture with MKs for 5 days (n=6 per group). (**D**) Pre-OBs were induced differentiation into mature OBs in osteogenic differentiation medium without or with MKs-CM from WT mice after 7 days. Representative alkaline phosphatase staining images (left) and quantification of the activity of alkaline phosphatase was calculated (right) (n=6 per group). (**E**) Pre-OBs were induced differentiation into mature OBs in osteogenic differentiation medium without or with MKs-CM from WT mice after 21 days. Representative Alizarin red staining images (left) and quantification of matrix mineralization was calculated (right) (n=6 per group). (**F**) Representative immunostaining images of CD31 (red) and Emcn (green) in the BM (left) and endocortical bone surfaces (right) of MK^deleted^ mice and their littermate controls. The quantification of CD31^hi^ Emcn^hi^ (yellow) cells are shown in the right panel (n=6 mice per group). Scale bar, 100 µm. (**G**) Representative images of tube formation of ECs (with or without co-cultures of OBs) after addition of MKs-CM. The quantitative analysis of cumulative tube length is shown in the right panel (n=6 per group). Scale bar, 100 µm. Data are shown as mean ± SD. *P < 0.05, **P < 0.01, ***P < 0.001. For all panels in this figure, data are representative of three independent experiments.

We next evaluated whether MKs affect the biological behavior of OBs. Indeed, deletion of MKs decreased the number of osteocalcin^+^ OBs on the trabecular (about 32.1%) and endocortical bone surfaces (about 51.2%) and reduced the osteocalcin levels in the BM (about 17.7%) and serum (about 20.8%) (Figure 2A, B). Moreover, we isolated and purified the primary BM-derived MKs, and found that either direct or indirect coculture of OBs with MKs significantly induced OBs proliferation and differentiation, suggesting that MKs-secreted factors play important roles in these processes (Figure 2C-E; Figure S2A, B).

A blood supply is essential for bone formation. In fact, the majority of OBs and their progenitors are selectively adjacent to CD31^hi^Emcn^hi^ vessels 5. Specifically, immunofluorescence staining revealed that the number of CD31^hi^Emcn^hi^ cells was reduced by approximately 64.8% on trabecular bone (TB) and by approximately 70% on endocortical bone (EB) in the distal femur of MK^deleted^ mice (Figure 2F). In addition, MKs-CM induced tube formation of vascular ECs (Figure 2G), although the proliferation of ECs was unchanged in vitro (Figure S2C). These results suggest that MKs not only promote the proliferation and differentiation of OBs but also facilitate ECs tube formation. Interestingly, the presence of OBs further enhanced MKs-mediated ECs tube formation (Figure 2G), suggesting that OBs and MKs have a synergistic effect.

### TGF-β1 secreted from BM MKs is sufficient to induce osteogenesis

**Figure 3:**
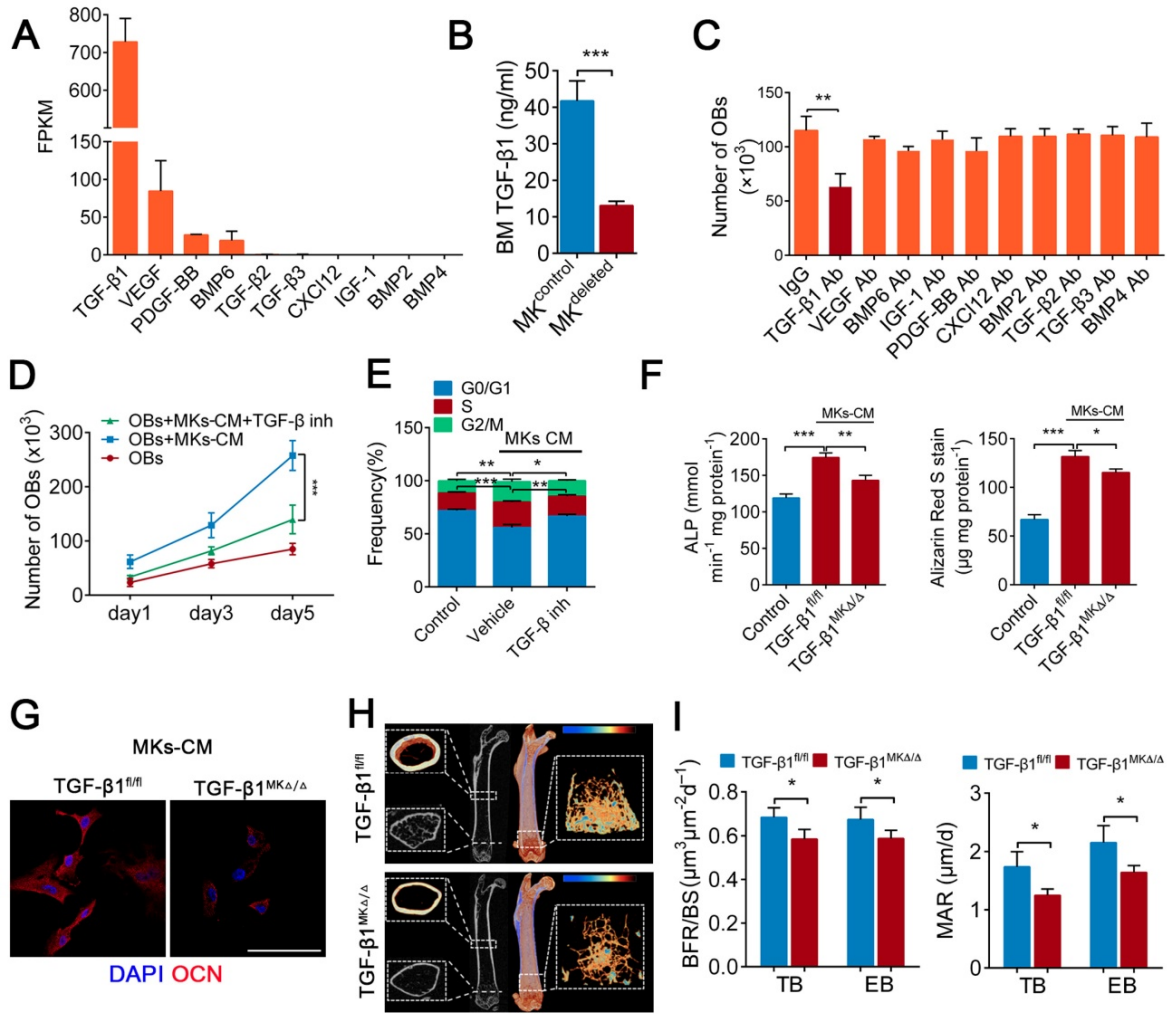
** TGF-β1 secreted from BM MKs is sufficient to induce osteogenesis.** (**A**) RNA-seq analysis of niche factors related to bone formation in human MKs. Expression levels are shown in terms of FPKM (fragments per kilobase of exon per million fragments mapped). (**B**) The concentrations of TGF-β1 in the BM of MK^deleted^ mice and their littermate controls, determined by ELISA (n=6 mice per group). (**C**) Proliferation of OBs in culture in the presence of MKs-CM and indicated individual neutralizing antibody (Ab) or IgG (n=6 per group). (**D**) Proliferation of OBs (pretreated TGF-β type I receptor inhibitor SB431542) in culture with MKs-CM for 5 days (n=6 per group). Inh, inhibitor. (**E**) Cell cycle analysis of OBs culture with MKs-CM for 3 days by flow cytometry (n=6 per group). Inh, inhibitor. (**F**) Pre-OBs were induced differentiation into mature OBs in osteogenic differentiation medium without or with MKs-CM from WT mice. Quantification of the activity of alkaline phosphatase (left) on day 7 and matrix mineralization on day 21 (right) (n=6 per group). (**G**) Representative immunostaining images of osteocalcin (red) OBs in osteogenic differentiation medium with MKs-CM from TGF-β1^MK∆/∆^ and TGF-β1^fl/fl^ mice after 14 days (n=6 per group). Scale bar, 100 µm. (**H**) Representative Micro-CT images of femur from TGF-β1^MK∆/∆^ and TGF-β1^fl/fl^ mice (n=6 mice per group). (**I**) Quantification of BFR and MAR in TB and EB of TGF-β1^MK∆/∆^ and TGF-β1^fl/fl^ mice (n=6 mice per group). Data are shown as mean ± SD. *P < 0.05, **P < 0.01, ***P < 0.001. For all panels in this figure, data are representative of three independent experiments.

A previous study found that MKs are the main source of TGF-β1 in the BM 20. Further, our RNA-seq data showed that the expression of TGF-β1 was higher than that of other known factors related to bone formation in MKs (Figure 3A), which was consistent with the results from ELISAs (Figure S3A). Indeed, the TGF-β1 concentration was reduced by approximately 68.6% in the BM of the MK^deleted^ mice (Figure 3B). We next assessed whether the MKs-derived TGF-β1 is involved in OBs proliferation and differentiation**.** Notably, only the neutralizing antibody against TGF-β1 significantly weakened MKs-CM induced OBs proliferation (Figure 3C), which was confirmed by a TGF-β type I receptor inhibitor (SB431542) (Figure 3D, E). The neutralizing antibody against TGF-β1 also abrogated the differentiation of OBs mediated by MKs (Figure S3B).

To further verify the effect of TGF-β1 secreted by MKs on OBs, we specifically deleted TGF-β1 in MKs by crossing Pf4-cre mice with TGF-β1^fl/fl^ mice. The TGF-β1 concentration was significantly decreased in the BM of Pf4-cre^+^; TGF-β1^fl/fl^ (hereafter referred to as TGF-β1^MK∆/∆^) mice (Figure S3C). Notably, the proliferation and differentiation of the OBs were reduced after the addition of MKs-CM from the TGF-β1^MK∆/∆^ mice compared with that from the control mice (Figure 3F, G; Figure S3D, E). More importantly, the TGF-β1^MK∆/∆^ mice exhibited an overall decrease in bone remodeling (Figure 3H), trabecular bone parameters and cortical bone thickness (Figure S3F). We found that the TGF-β1^MK∆/∆^ mice had decreased bone formation and reduced bone strength and stiffness (Figure 3I; Figure S3G-I). Taken together, these findings suggest that TGF-β1 derived from MKs induces osteogenesis in mice.

### TGF-β1 from MKs induces angiogenesis via HIF-1α

**Figure 4:**
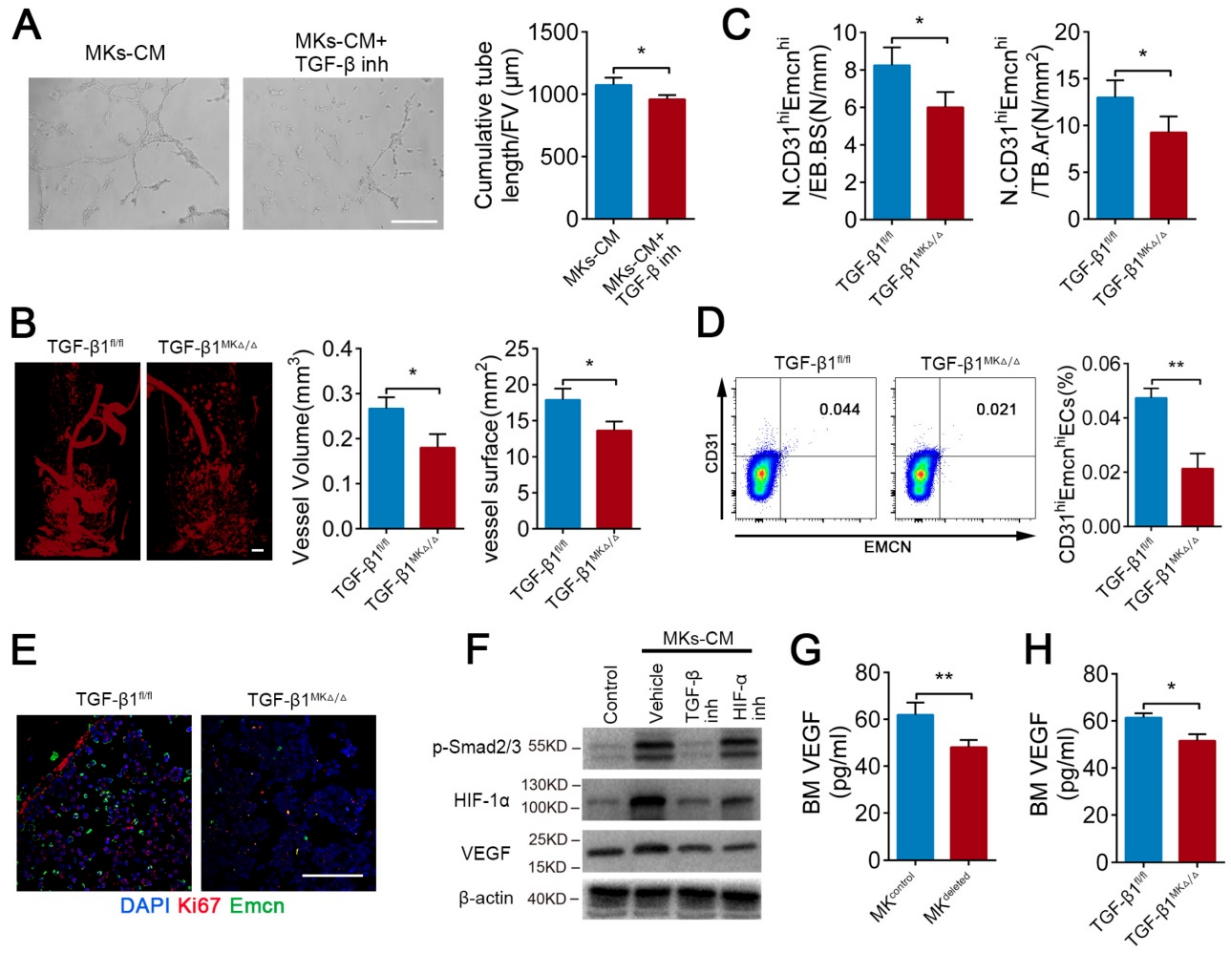
** TGF-β1 from MKs induce angiogenesis via HIF-1α.** (**A**) Representative images of tube formation of ECs (pre-treated TGF-β inhibitor SB431542) after addition of MKs-CM (n=6 per group). The quantitative analyses of cumulative tube length are shown in the right panel. Scale bar, 100 µm. Inh, inhibitor. (**B**) Angiographic analysis of the volume and surface area of bone vessels from TGF-β1^MK∆/∆^ and TGF-β1^fl/fl^ mice (n=6 mice per group). Representative angiographic images are shown in the left panel. Scale bar, 1 mm. (**C**) The quantification of CD31^hi^ Emcn^hi^ cells in the BM of TGF-β1^MK∆/∆^ and TGF-β1^fl/fl^ mice (n=6 mice per group). (**D**) Flow cytometric analysis of the percentage of CD31^hi^ Emcn^hi^ ECs in the BM of TGF-β1^MK∆/∆^ and TGF-β1^fl/fl^ mice (n=6 mice per group). (**E**) Representative immunostaining images of Emcn (red) and Ki67 (green) in the BM of TGF-β1^MK∆/∆^ and TGF-β1^fl/fl^ mice (n=6 mice per group). Scale bar, 100 µm. (**F**) Western blot analysis of the expressions of p-smad2/3, HIF-1α and VEGF in ECs (pretreated with TGF-β or HIF-1α inhibitor) after addition of MKs-CM (n=6 per group). (**G**) The concentration of VEGF in the BM of MK-deleted mice and their littermates, determined by ELISA (n = 6 mice per group). (**H**) The concentration of VEGF in the BM of TGF-β1^MK∆/∆^ and TGF-β1^fl/fl^ mice, determined by ELISA (n=6 mice per group). Data are shown as mean ± SD. *P < 0.05, **P < 0.01. For all panels in this figure, data are representative of three independent experiments.

It has been well established that TGF-β1 can promote angiogenesis. As anticipated, MKs-induced ECs tube formation was weakened by approximately 10.6% after pretreatment with TGF-β inhibitor (SB431542) (Figure 4A). Microfil-perfused angiography revealed that the vessel volume was reduced 32.5% and vessel surface area was reduced 23.8% in the TGF-β1^MK∆/∆^ mice compared with the control mice (Figure 4B). Moreover, the TGF-β1^MK∆/∆^ mice displayed a significant decrease in the number of CD31^hi^Emcn^hi^ ECs in the BM, accompanied by decreased proliferation of ECs (Figure 4C-E). Therefore, these results suggest that MKs-secreted TGF-β1 promotes angiogenesis.

The endosteal niche is always a low oxygen environment, and HIF-1α controls angiogenesis under physiological and pathological conditions. However, the relationship between TGF-β1 and HIF-1α in bone-related angiogenesis is unknown. Intriguingly, we found that MKs-CM induced the expression of HIF-1α in ECs in a SMAD2/3-dependent way (Figure 4F). In addition, both TGF-β inhibitor (SB431542) and HIF-1α inhibitor (oltipraz) reduced the level of VEGF, which is a critical cytokine in promoting angiogenesis 47, in ECs (Figure 4F). Consistent with these findings, the VEGF concentration was evidently reduced by approximately 22.3% and 15.9% in the BM of the MK^deleted^ and TGF-β1^MK∆/∆^ mice, respectively (Figure 4G, H). The findings above indicate that MKs-derived TGF-β1 can enhance the expression of VEGF in ECs by up-regulating HIF-1α expression, eventually controlling bone-related H-type angiogenesis.

### MKs attenuate irradiation-induced bone loss in mice by secreting TGF-β1

**Figure 5:**
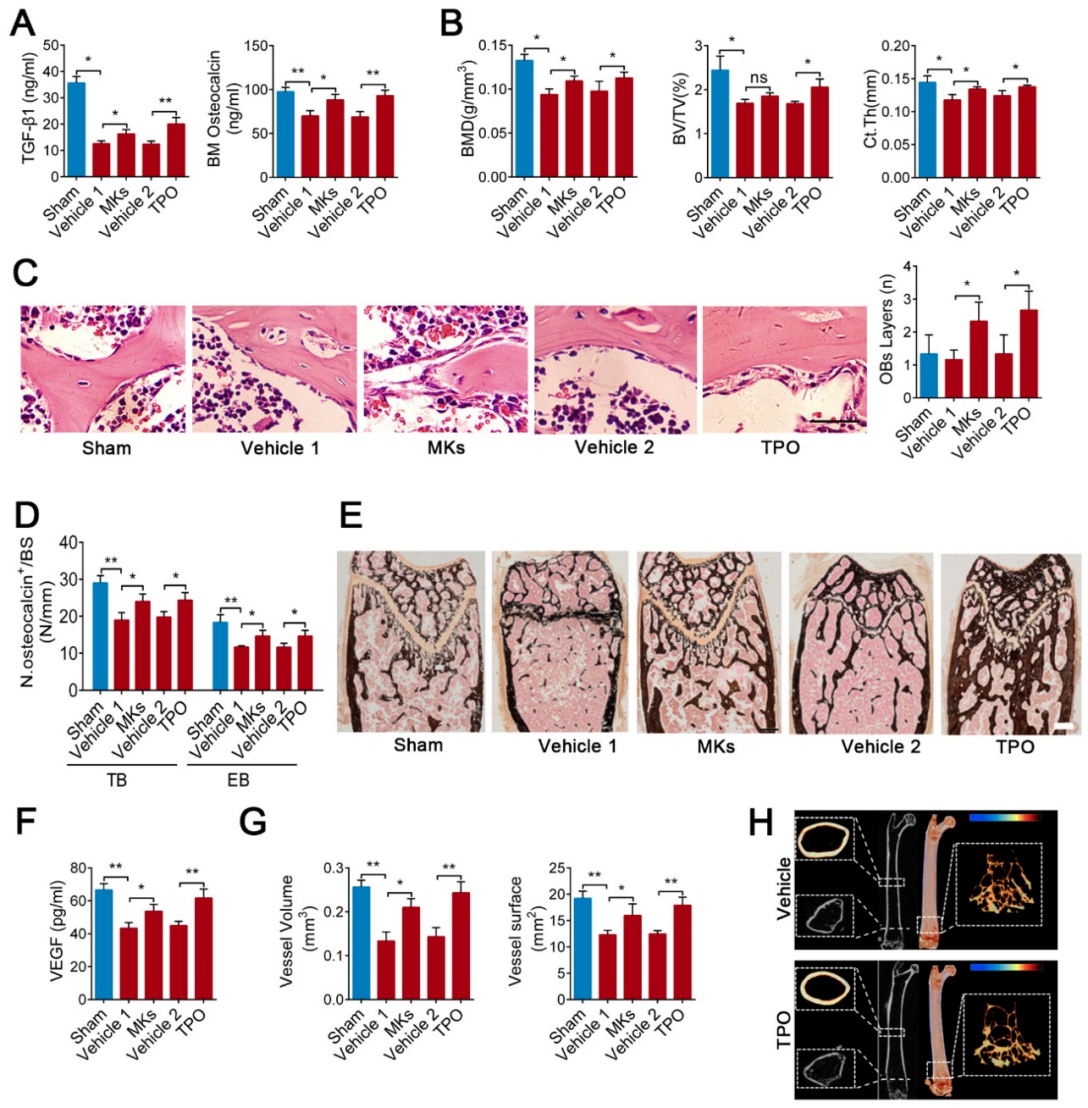
** MKs attenuate irradiation-induced bone loss in mice by secreting TGF-β1.** (**A**) The concentrations of TGF-β1 and osteocalcin in the BM of sham or irradiated mice 2 months after treated with MKs or TPO, determined by ELISA (n=6 mice per group). (**B**) Quantitative Micro-CT analysis of the BMD, BV/TV and Ct.Th of femora from sham or irradiated mice 2 months after treated with MKs or TPO (n=6 mice per group). (**C**) HE staining demonstrating the layers of endosteal osteoblasts in the BM from sham or irradiated mice 2 weeks after treated with MKs or TPO (n=6 mice per group). Scale bar, 200 µm. (**D**) Quantification of osteocalcin^+^ cell numbers on surface of TB and EB from sham or irradiated mice 2 months after treated with MKs or TPO (n=6 mice per group). (**E**) Von Kossa staining showing the mineralization of bone matrix in sham or irradiated mice 2 months after treated with MKs or TPO (n=6 mice per group). Scale bar, 50 µm. (**F**) The concentration of VEGF in the BM of sham or irradiated mice 2 months after treated with MKs or TPO, determined by ELISA (n=6 mice per group). (**G**) Angiographic analysis of the volume and surface area of bone vessels from sham or irradiated mice 2 months after treated with MKs or TPO (n=6 mice per group). (**H**) Representative Micro-CT images of longitudinal section femurs, cross-sectional view of the distal femurs and reconstructed trabecular structure of the ROI (White dashed box) in TGF-β1^MK∆/∆^ mice with or without radioactive bone injury 2 months after treatment of TPO. Color scale bar represents bone mineral density level. (n=6 mice per group). Data are shown as mean ± SD. *P < 0.05, **P < 0.01. ns, no significant. For all panels in this figure, data are representative of three independent experiments.

Irradiation-induced osteoporosis is associated with decreased bone formation and angiogenesis. Next, we assessed whether increasing MKs abundance can alleviate irradiation-induced osteoporosis. Local injection of MKs significantly increased the TGF-β1 and osteocalcin concentrations in the BM of mice with irradiative bone injury, according with the effect of systemic delivery of TPO (Figure 5A). Further investigations showed that treatment with MKs or TPO increased bone formation and strength in irradiated mice (Figure 5B-E; Figure S4A-E). However, we discovered that the augment of MKs number significantly increased VEGF concentrations in the BM, as well as angiogenesis, in irradiated mice (Figure 5F, G; Figure S4F, G).

We then investigated whether MK-induced attenuation of irradiation-induced bone loss is mediated through secretion of TGF-β1. Although TPO injection evidently increased the MKs number in the TGF-β1^MK∆/∆^ mice after irradiation, the bone loss was not alleviated (Figure 5H; Figure S4H). The BM VEGF concentration was only slightly increased, and angiogenesis was not significantly elevated in the irradiated TGF-β1^MK∆/∆^ mice after TPO treatment (Figure S4I-K). Thus, MKs-derived TGF-β1 plays a key role in promoting bone formation and bone-related angiogenesis after irradiation.

### MKs relieve irradiation-induced apoptosis of OBs via DNA damage repair

**Figure 6:**
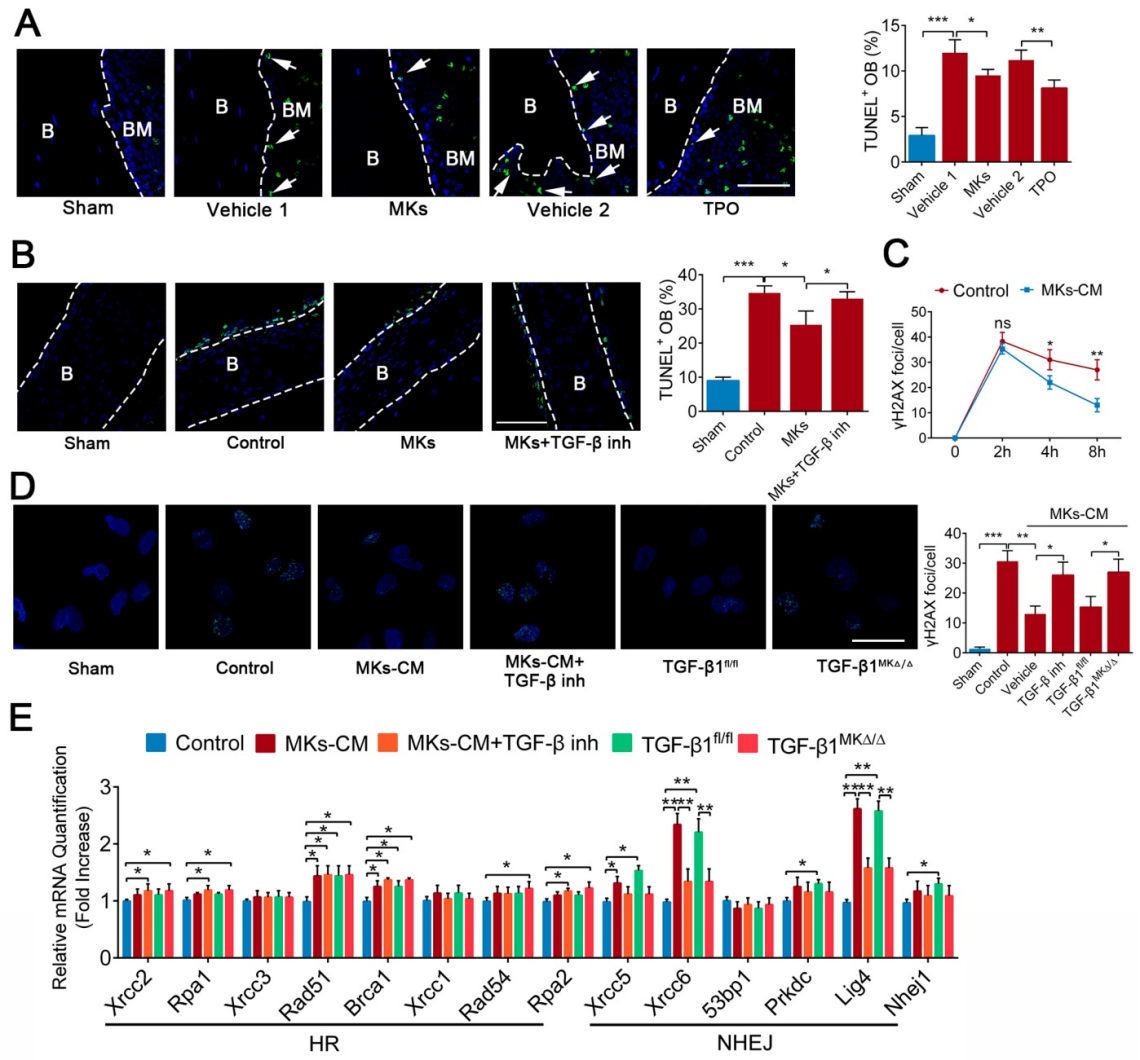
** MKs relieve radiation-induced apoptosis of OBs via repairing DNA damage.** (**A**) Representative images of TUNEL staining on femur from sham or irradiated mice 2 weeks after treated with MKs or TPO. The quantification of TUNEL positive OBs are shown in the right panel (n=6 mice per group). Dashed lines outline bone surface. Scale bar, 100 µm. B, bone. BM, bone marrow. (**B**) Calvariaes harvested from neonatal mouse pups were subjected to 16 Gy irradiation, and then treated with vehicle, MKs or MKs+TGF-β inhibitor. Twenty-four hours later, TUNEL staining was used to detect the apoptosis of OBs on these calvariaes (n=6 mice per group). Scale bar, 100 µm. B, bone. BM, bone marrow. Inh, inhibitor. (**C**) Immunofluorescence staining of γ-H2AX in OBs treated with or without of MKs-CM 2, 4 and 8 hours after radiation. The quantitation of the numbers of γ-H2AX foci per cells are shown in the right panel (n=6 per group). Note that MKs-CM was added immediately post-radiation. (**D**) Immunofluorescence staining of γ-H2AX in OBs from control, MKs+vehicle, MKs+ TGF-β inhibitor, MKs (from TGF-β1^fl/fl^ mice) and MKs (from TGF-β1^MK∆/∆^ mice) groups 24 hours after 12 Gy irradiation (n=6 per group). Scale bar, 100 µm. (**E**) Quantitative PCR analysis of the expressions of DNA repair-associated genes in OBs from control, MKs+vehicle, MKs+TGF-β inhibitor, MKs (from TGF-β1^fl/fl^ mice) and MKs (from TGF-β1^MK∆/∆^ mice) groups 24 hours after 12 Gy IR exposure (n=6 per group). Data are shown as mean ± SD. *P < 0.05, **P < 0.01, ***P < 0.001. For all panels in this figure, data are representative of three independent experiments.

Given that apoptosis and DNA damage also contribute to the impairment of bone formation after irradiation, we investigated whether MKs can affect irradiation-induced damage in OBs. TUNEL staining showed that systemic delivery of TPO or local injection of MKs in mice protected OBs from cell apoptosis induced by irradiation (Figure 6A). To further confirm this idea, we used newborn mouse calvarial bone, which retains the bone structure and more closely approximates physiological conditions than cell lines. Specifically, MKs decreased the apoptotic cells on the calvarial surface after irradiation, as shown by TUNEL staining, compared with the vehicle (Figure 6B), and these results were further confirmed by cleaved caspase-3 staining (Figure S5A). Similar results were obtained in primary OBs by flow cytometric analysis (Figure S5B). In particular, TGF-β inhibitor (SB431542) significantly blocked the anti-apoptotic effect of MKs, and MKs from the TGF-β1^MK∆/∆^ mice could not effectively inhibit the irradiation-induced apoptosis of OBs (Figure S5B), suggesting that MKs alleviation of irradiation-induced OBs apoptosis is at least in part attributed to TGF-β1.

Double strand breaks (DSBs) is the most serious form of irradiation-induced DNA damage. Specifically, we found that MKs-CM significantly promoted DNA damage repair, as shown by γ-H2AX staining, although the DNA formation was unchanged after irradiation exposure (Figure 6C, D; Figure S5C). Irradiation-induced DSBs are repaired through the homologous recombination (HR) and non-homologous end-joining (NHEJ) pathways. Importantly, the expressions of the DNA repair-associated genes (Rad51, Brca1, Xrcc5, Xrcc6 and Lig4) were significantly upregulated in the OBs in the presence of MKs-CM. However, this effect was mostly abrogated by TGF-β inhibition or deficiency (Figure 6E). Altogether, our results showed that TGF-β1 produced by MKs can significantly suppress apoptosis and induce DSBs repair in OBs after irradiation.

## Discussion

**Figure 7:**
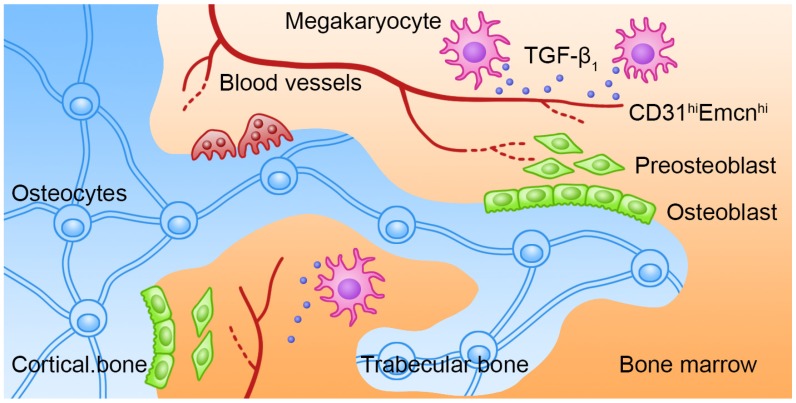
** Schematic illustration of the role of MKs-derived TGF-β1 in promoting bone formation.** During cortical bone and trabecular bone remodeling, MKs secret TGF-β1 to promote osteoblasts proliferation and differentiation and induce the formation of CD31^hi^Emcn^hi^ vessels.

Bone remolding is a complicated process that is controlled by the balance of OCs-induced bone resorption and OBs-induced bone formation. The biological behaviors of OBs are coordinately regulated by several intrinsic and BM niche-derived factors. However, how MKs regulate bone formation under physiological and pathological conditions has not been fully elucidated. In our study, we showed that BM MKs can secret TGF-β1 to promote bone formation through coupling osteogenesis with angiogenesis (Figure 7).

It has been well established that c-Mpl is a critical gene for megakaryocytopoiesis, and systemic knockout of c-Mpl leads to a strong decrease in MKs number in the mouse BM 32. Interestingly, because c-Mpl can intrinsically inhibit the proliferation of OBs, c-Mpl inactivation leads to an increased trabecular bone mass during aging 31. Nevertheless, the thickness and strength of the cortical bone decreased in mice when c-Mpl was deleted 31, which may be due to the decrease in MKs number in the BM. To exclude the direct role of c-Mpl in OBs, we performed a transplantation assay. Notably, the mice reconstituted with c-Mpl^-/-^ spleen cells exhibited a significant decrease in bone mass and strength, indicating that MKs affect bone remolding. We then used a megakaryocyte specific Cre (Pf4-Cre) mouse model. Although a low-level of ectopic recombination of Pf4 is present 48, it is not significantly induced under our experimental conditions (Figure S1A-C). Thus, this mouse model remains a good available tool for megakaryocyte-associated investigations. As a result, targeted deletion of MKs suppressed bone formation in mice. MKs are a very important source of cytokines associated with bone remolding in the BM niche 24, 49-51. Indeed, we found that MKs-CM significantly promoted MKs proliferation and differentiation. Further investigations revealed that TGF-β1 was highly enriched in MKs and that ablation of MKs reduced the TGF-β1 concentration in the mouse BM. TGF-β1 inhibition significantly suppressed the proliferation and differentiation of the OBs induced by MKs-CM. More importantly, conditional deletion of TGF-β1 in MKs reduced bone formation as well as bone strength and stiffness in vivo. Based on these combined data, we believe that TGF-β1 plays a main role in MKs-mediated proliferation and differentiation of OBs. However, there are controversial views on whether MKs can promote or inhibit OBs differentiation 52, 53. Indeed, our data indicated that MKs can promote the differentiation of OBs by secreting TGF-β1. It was well accepted that TGF-β1 has a bidirectional effect on differentiation in vitro, which is dependent on the TGF-β1 concentrations and different stages of OBs 54, 55.

The formation of blood vessels influences the microenvironment during osteoprogenitors differentiation 56, 57. Recently, one study reported that the CD31^hi^Emcn^hi^ vessel subtype has specific molecular and morphological characteristics and locations, which are associated with bone formation 5. In the present study, we confirmed by in vitro and in vivo experiments that MKs-derived TGF-β1 promotes angiogenesis in the BM by inducing the formation of CD31^hi^Emcn^hi^ vessels. HIF-1α is a cellular oxygen sensor that can be induced by a low oxygen environment 5. HIF-1α controls both physiological and pathological neovascularization 58. In addition, HIF-1α can upregulate the expression of VEGF and accelerate wound healing in ECs 59. Here, we demonstrated that the expression levels of both HIF-1α and VEGF are elevated in cultured ECs after addition of MKs-CM from the TGF-β1^fl/fl^ mice, but not the TGF-β1^MK∆/∆^ mice. These data are in line with a previous study showing that TGF-β1 further enhanced hypoxia-stimulated HIF-1α expression in renal epithelial cells 60. Furthermore, our data revealed that MKs can secret VEGF, which may directly participate in bone-related angiogenesis. Thus, MKs are involved in OBs proliferation, differentiation and angiogenesis in the BM, which is at least partly mediated by TGF-β1.

Ionizing irradiation induces the suppression of bone formation and activation of bone absorption, eventually resulting in radioactive bone loss. Indeed, bone vessels have been shown to be sensitive to ionizing irradiation, which can cause endarteritis and occlusion of the blood vessels 10. Subsequently, the occlusion of bone vessels results in severe hypoxia and malnutrition of the stromal cells and ECs in the BM. In this work, we showed that TGF-β1 secreted by MKs facilitates the formation of temporospatial vessels that are needed for new bone formation after irradiation. However, although OBs are insensitive to irradiation, high doses of local irradiation can still cause drastic damage to OBs. We found that MKs can significantly alleviate irradiation-induced damage in OBs by secreting TGF-β1. The exact role of TGF-β1 in irradiation is still a matter of debate, with some studies showing that it acts as a radiosensitizer and others reporting that it functions as a radioprotector 61, 62. In our study, we showed that TGF-β1 is a radioprotector of OBs by reducing apoptosis and accelerating DNA damage repair. Moreover, MKs-derived TGF-β1 promotes DNA damage repair mainly by increasing NHEJ activity. Nevertheless, how TGF-β1 increases the expressions of DNA damage repair-associated genes require further research. Taken together, the reduction of CD31^hi^Emcn^hi^ vessels and the direct damage of OBs may provide a reasonable explanation for radioactive osteoporosis, which can be relieved by MKs-derived TGF-β1.

In conclusion, our findings demonstrate that MKs play a crucial role in bone formation through promoting osteogenesis and angiogenesis at least in part by secreting TGF-β1. In addition, this study suggests that MKs-induced bone formation provides a potential therapeutic target for osteoporosis induced by irradiation or other pathological factors. Consequently, given its role in indirectly improving bone formation while promoting hematopoietic recovery, systemic intermittent delivery of TPO might be a suitable treatment for radiotherapy-caused complications.

## Supplementary Material

Supplementary materials and methods, figures, and table.

## Abbreviations

MKs: megakaryocytes
OBs: osteoblasts
OCs: osteoclasts
ECs: endothelial cells
CM: conditioned medium
BMD: bone mineral density
BV/TV: trabecular bone volume fraction
Tb.N: trabecular number
Tb.Th: trabecular thickness
Tb.Sp: trabecular separation
Ct. Th: cortical thickness
Ob.S/BS: the percentage of trabecular bone surface covered by osteoblasts
Ob.N/B.Pm: osteoblast number/bone perimeter
inh: inhibitor
TPO: thrombopoietin
DSBs: Double strand sreaks
HR: homologous recombination
NHEJ: non-homologous end-joining
