# Supplementary material for: Megakaryocytes promote bone formation through coupling osteogenesis with angiogenesis by secreting TGF-β1
Source: Theranostics. 2020 Jan 12;10(5):2229–42. doi: 10.7150/thno.40559 (PMC7019172; doi:10.7150/thno.40559)
Supplement: Supplementary file 1 — Supplementary materials and methods, figures, and table. [file thnov10p2229s1.pdf]

Megakaryocytes promote bone formation through coupling osteogenesis with angiogenesis by secreting TGF-β1

Yong Tang[1, *], Mengjia Hu[1, *], Yang Xu[1], Fang Chen[1], Shilei Chen[1], Mo Chen[1], Yan Qi[1], Mingqiang Shen[1], Cheng Wang[1], Yukai Lu[1], Zihao Zhang[1], Hao Zeng[1], Yong Quan[1], Fengchao Wang[1], Yongping Su[1], Dongfeng Zeng[1], Song Wang[1, #], Junping Wang[1, #].

Supplemental information

Materials and Methods

Mice

CAG-LoxP-ZsGreen-Stop-LoxP-tdTomato (Rosa26-mT/mG) mice were purchased from Nanjing biomedical research institute of Nanjing University (Nanjing, China). Pf4-cre^{+}; Rosa26-mT/mG mice were generated by crossing Rosa26-mT/mG mice with Pf4-cre^{+} mice. Littermate Pf4-cre^{-}; Rosa26-mT/mG mice were served as negative controls.

DT injection and irradiation.

Adult Pf4-cre^{+}; Rosa26-mT/mG mice were injected with DT (at the dose of 50 ng/g body weight) every two days. Two weeks after first injection, these mice were used for subsequent analysis. In addition, adult Pf4-cre^{+}; Rosa26-mT/mG mice were subjected to 6.5 Gy and 3.5 Gy irradiation at day 1 and day 14, respectively. Four weeks after first irradiation, these mice were used for subsequent analysis.

Preparation of macrophages, fibroblasts and OBs.

BM macrophages were labeled with anti-mouse CD11b (M1/70; Biolegend) and F4/80 (BM8; Biolegend) antibodies.

BM-specific fibroblasts were isolated as described [1]. Briefly, BM cells were flushed from femur and tibia of mice. Bone marrow Mononuclear (BMM) cells were then isolated using Lympholyte-M (Cedarlane, Hornby, Canada) gradient centrifugation, and resuspended in RPMI-1640 (Hyclone, Logan, Utah, USA) medium supplemented with penicillin/streptomycin, glutamine, minimum essential medium and sodium pyruvate. Subsequently, BM stromal cells were obtained after the adhesion of BMM cells to polystyrene flasks and cultured in DMEM medium (Hyclone) containing 10% fetal bovine serum (FBS; Hyclone). Fibroblasts were purified from BM stromal cells by MACS using anti-fibroblast marker (sc-73355, Santa cruz, Rat IgG) in combination with anti-Rat IgG MicroBeads (Miltenyi Biotec), followed by flow cytometric analysis.

BM-derived mature OBs were isolated as described [2]. BM cells were flushed from the femur and tibia of mice. Cells were centrifuged at 300g for 10 min and re-suspended in 200 μL of ice-cold buffer (Dulbecco's phosphate buffered saline without Ca^{2+} and Mg^{2+}, with 0.5% bovine serum albumin and 2 mM EDTA). The mature OBs (ALP^{+} cells) were purified by MACS using anti-ALP antibody (ab108337, Rabbit IgG, Abcam) in combination with anti-Rabbit IgG MicroBeads (Miltenyi Biotec), followed by flow cytometric analysis.

Reference

[1] Frassanito MA, Rao L, Moschetta M, Ria R, Di Marzo L, De Luisi A, et al. Bone marrow fibroblasts parallel multiple myeloma progression in patients and mice: in vitro and in vivo studies. Leukemia. 2014; 28: 904-16.

[2] Li D, Liu J, Guo B, Liang C, Dang L, Lu C, et al. Osteoclast-derived exosomal miR-214-3p inhibits osteoblastic bone formation. Nat Commun. 2016; 7: 10872.

Figure S1

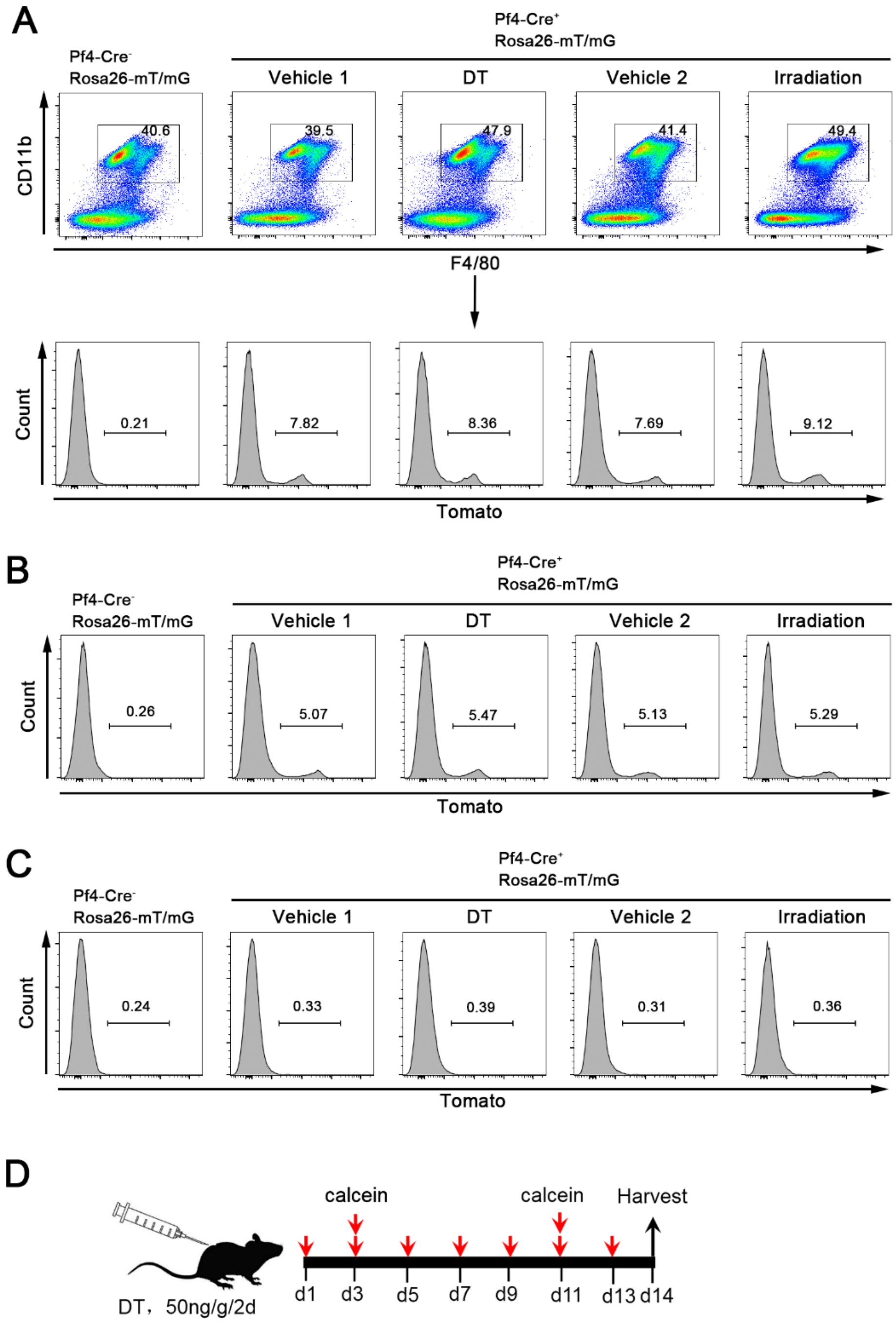

Pf4-Cre is ectopic recombined at a low-level in macrophages, fibroblasts and osteoblasts under normal conditions and in the context of DT injection or irradiation. (**A-C**) Flow cytometric analysis of

the percentage of Tomato-expressing cells in (A) macrophages ($CD11b^+$, $F4\text{-}80^+$), (**B**) fibroblasts and (C) osteoblasts obtained from the BM of adult Pf4-cre$^+$; Rosa26-mT/mG mice after DT injection or irradiation (Vehicle 1 vs DT, vehicle 2 vs irradiation). Pf4-cre$^-$; Rosa26-mT/mG littermates were served as negative controls. Data are representative of three independent experiments. (D) Scheme for DT administration to Pf4-cre$^+$;iDTR and Pf4-cre$^-$;iDTR mice.

Figure S2

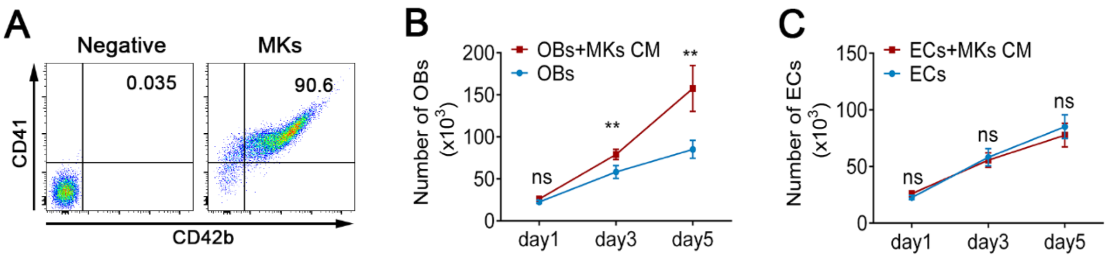

MKs promote OBs proliferation, but have no significant effect on ECs proliferation invitro. (A) The purity of megakaryocytes on 9th day after culture was about 90.6%, which was determined by flow cytometry according to the expressions of CD41 and CD42b. (**B**) Proliferation of OBs in indirect culture with or without MKs-CM for 5 days (n=6 per group). (**C**) Proliferation of ECs in indirect culture with or without MKs-CM for 5 days (n=6 per group). Data are shown as mean ± SD. **P < 0.01. ns, no significant. For all panels in this figure, data are representative of three independent experiments.

Figure S3

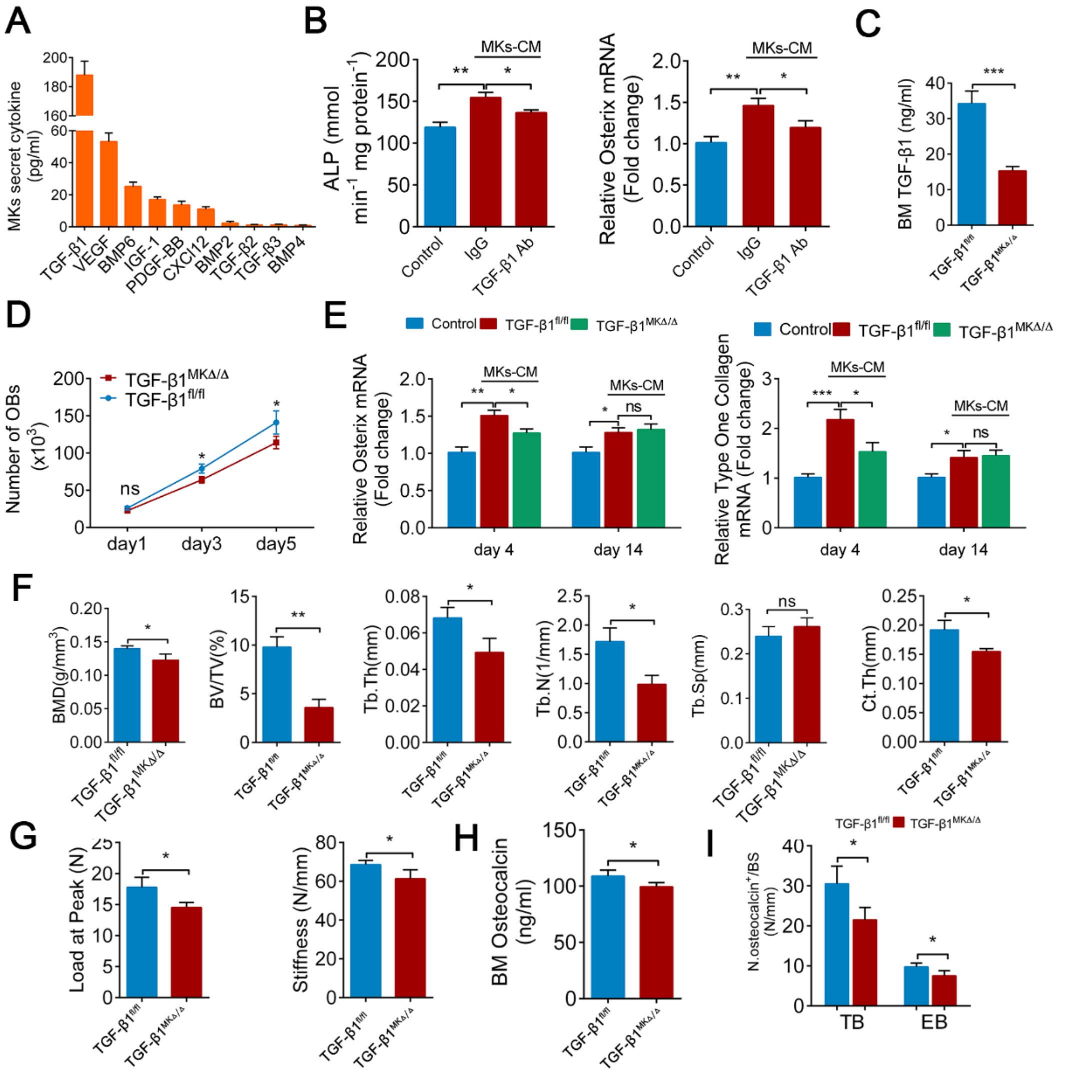

TGF-β1 secreted by MKs promotes the proliferation and differentiation of osteoblasts in vitro and bone formation in vivo. (**A**) The concentrations of TGF-β1, VEGF, BMP6, IGF-1, PDGF-BB, CXCl12, BMP2, TGF-β2, TGF-β3 and BMP4 in MKs-CM determined by ELISA (n=6 per group). (**B**) Differentiation of OBs in culture in the presence of MKs-CM and indicated individual neutralizing antibody (Ab) or IgG. Quantification of the activity of alkaline phosphatase (left) and (right) on day 7 (n=6 per group). (**C**) The concentration of TGF-β1 in the BM of TGF-$\beta1^{MK\Delta/\Delta}$ and TGF-$\beta1^{fl/fl}$ mice, determined by ELISA (n=6 mice per group). (**D**) Proliferation of OBs in culture with MKs-CM from TGF-$\beta1^{MK\Delta/\Delta}$ and

TGF-$\beta 1^{fl/fl}$ mice for 5 days (n=6 per group). (**E**) Relative mRNA level of osteorix and type I collagen during differentiation of OBs treated without or with MKs-CM from TGF-$\beta 1^{MK\Delta/\Delta}$ and TGF-$\beta 1^{fl/fl}$ mice for 4 days and 14 days (n=6 per group). (**F**) Quantitative Micro-CT analysis of BMD, BV/TV, Tb.N, Tb.Th, Tb.Sp and Ct.Th of femur from TGF-$\beta 1^{MK\Delta/\Delta}$ and TGF-$\beta 1^{fl/fl}$ mice (n=6 mice per group). (**G**) Quantitative biomechanical analysis of femur (Load of peak and stiffness) from TGF-$\beta 1^{MK\Delta/\Delta}$ and TGF-$\beta 1^{fl/fl}$ mice (n=6 mice per group). (**H**) Bone marrow osteocalcin concentrations by ELISA from TGF-$\beta 1^{MK\Delta/\Delta}$ and TGF-$\beta 1^{fl/fl}$ mice (n=6 mice per group). (**I**) The quantification of osteocalcin^{+} cells on the surfaces of TB and EB from TGF-$\beta 1^{MK\Delta/\Delta}$ and TGF-$\beta 1^{fl/fl}$ mice (n=6 mice per group). Data are shown as mean ± SD. *P < 0.05, **P < 0.01, ***P < 0.001. ns, no significant. (Student's t-test). For all panels in this figure, data are representative of three independent experiments.

Figure S4

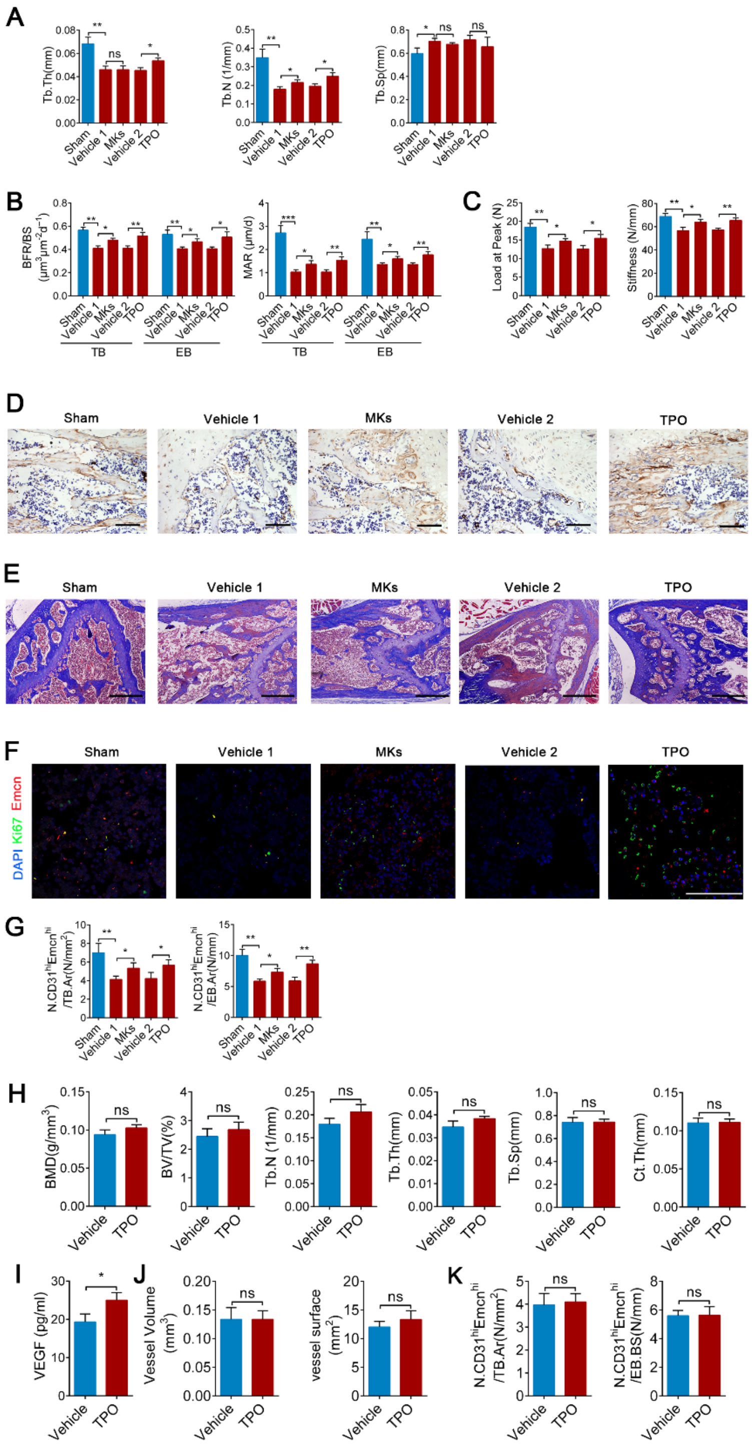

A
Tb.Th(mm)
Tb.N (1/mm)
Tb.Sp(mm)
Sham
Vehicle 1
MKs
Vehicle 2
TPO
ns
B
BFR/BS
MAR (μm/d)
TB
EB
C
Load at Peak (N)
Stiffness (N/mm)
D
E
F
DAPI Ki67 Emcn
G
H
BMD(g/mm3)
BV/TV(%)
Ct.Th(mm)
Vehicle
I
VEGF (pg/ml)
J
Vessel Volume
vessel surface
K

TGF-β1 secreted by MKs alleviates radioactive osteoporosis in mice by promoting bone formation. (**A**) Quantitative Micro-CT analysis of the trabecular bone fraction (Tb.N, Tb.Th and Tb.Sp) of femur from sham or irradiated mice 2 months after treated with MKs or TPO (n=6 mice per group). (**B**) The values of bone histomorphometry parameters (MAR, BFR) at the distal femur metaphysis from sham or irradiated mice 2 months after treated with MKs or TPO (n=6 mice per group). (**C**) Quantitative biomechanical analysis of femur (Load of peak and stiffness) from sham or irradiated mice 2 months after treated with MKs or TPO (n=6 mice per group). (**D**) Representative images of immunostaining of type I collagen on distal femur metaphysis from sham or irradiated mice 2 months after treated with MKs or TPO (n=6 mice per group). Scale bar, 100 μm. (**E**) Representative images of immunostaining of masson staining on distal femur metaphysis from sham or irradiated mice 2 months after treated with MKs or TPO (n=6 mice per group). Scale bar, 100 μm. (**F**) Representative images of Emcn (red) and Ki67 (green) immunostaining of proliferating endothelial cells from sham or irradiated mice 2 months after treated with MKs or TPO (n=6 mice per group). Scale bar, 100 μm. (**G**) The quantification of $CD31^{hi}$ $Emcn^{hi}$ cells in the BM of sham or irradiated mice 2 months after treated with MKs or TPO (n=6 mice per group). (**H**) Quantitative Micro-CT analysis of BMD, BV/TV, Tb.N, Tb.Th, Tb.Sp and Ct.Th of femur from TGF-$\beta1^{MK\Delta/\Delta}$ mice with or without radioactive bone injury 2 months after treated TPO (n=6 mice per group). (**I**) VEGF concentrations in bone marrow of TGF-$\beta1^{MK\Delta/\Delta}$ mice with or without radioactive bone injury 2 months after treated with TPO (n=6 mice per group). (**J**) Angiography-based quantification of vessel volume and surface area from TGF-$\beta1^{MK\Delta/\Delta}$ mice with or without radioactive bone injury 2 months after treated with TPO (n=6 mice per group). (**K**) Quantification of $CD31^{hi}Emcn^{hi}$ immunostaining of femur from TGF-$\beta1^{MK\Delta/\Delta}$ mice with or without radioactive bone injury 2 months after treated with TPO (n=6 mice per group). Data are shown as mean ± SD. *P < 0.05, **P < 0.01, ***P < 0.001. ns, no significant. (Student's t-test). For all panels in this figure, data are representative of three independent experiments.

Figure S5

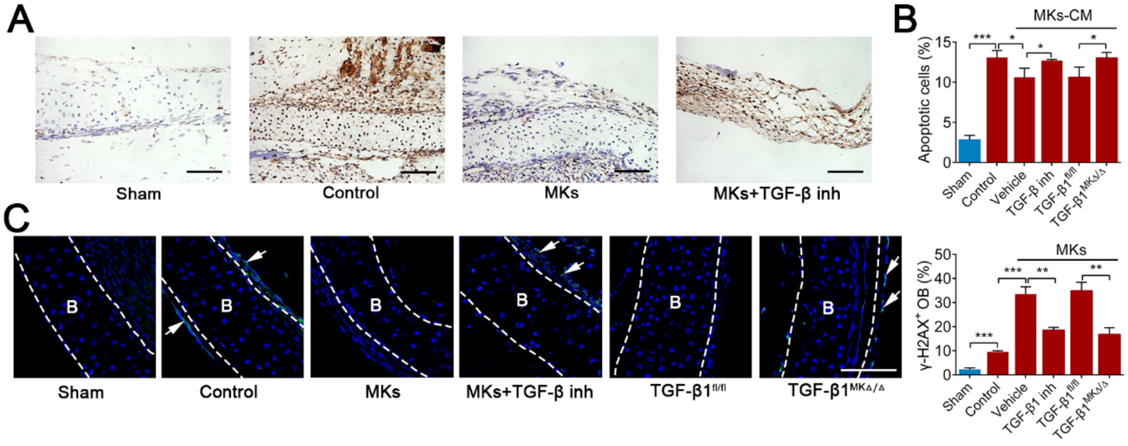

MKs can repair DNA damage and reduce apoptosis of OBs by secreting TGF-β1. (**A**) Representative images of cleaved-caspase-3 immunostaining of calvariae from sham or irradiation 24 hours after treated with MKs or MKs+TGF-β inhibitor (n=6 per group). Scale bar, 100 µm. Inh, inhibitor. (**B**) Flow cytometric analysis of the apoptosis of OBs in control, MKs-CM plus vehicle, MKs-CM plus TGF-β inhibitor, MKs-CM (from TGF-β1$^{fl/fl}$ mice) and MKs-CM (from TGF-β1$^{MK\Delta/\Delta}$ mice) groups 24 hours after 12 Gy irradiation (n=6 per group). (**C**) Calvariae harvested from neonatal mouse pups were irradiated and treated with MKs in growth medium with or without TGF-β inhibitor. Calvariae were harvested and followed by γ-H2AX staining after 12 hours. The percentage of apoptotic OBs in calvariae was quantified (n=6 mice per group). Scale bar, 100 µm. Dashed lines outline bone surface. Inh, inhibitor. Data are shown as mean ± SD. $^{**}P < 0.01$, $^{***}P < 0.001$. ns, no significant. (Student's t-test). For all panels in this figure, data are representative of three independent experiments.

Table S1

Primer sequences

**Gene**		**Sequence (5' -> 3')**
Osterix	Forward Primer	GGAAAGGAGGCACAAAGAAGC
	Reverse Primer	CCCCTTAGGCACTAGGAGC
Type I collagen	Forward Primer	GCTCCTCTTAGGGGCCACT
	Reverse Primer	ATTGGGGACCCTTAGGCCAT
Xrcc2	Forward Primer	ATGTGTAGCGACTTTCGCAGA
	Reverse Primer	CATCAGCAAACAGGTTGGGTT
Rapa1	Forward Primer	CAGTTCGCCAGTGGACTGAAG
	Reverse Primer	GCTGGTCATAGAAGCGAGTAGAC
Xrcc3	Forward Primer	CGAATTACTGCTGCGGTTAAGA
	Reverse Primer	CCCGAAGGTGTAGAGAGGCA
Rad51	Forward Primer	CGGGAGTTGGTGGGTTATCC
	Reverse Primer	CCGGCACATCTTGGTTTATTTGT
Brca1	Forward Primer	CTCCTGGTGGAAGATTTCGGT
	Reverse Primer	GAGTGGCACAAGAGTTGGGAA
Xrcc1	Forward Primer	AGCCAGGACTCGACCCATT
	Reverse Primer	CAAAGGCCGAGCCATCATTG
Rad54	Forward Primer	CCGGTGGTACGAGTCTTCG
	Reverse Primer	GATGGATTGCCTAAAGCCACAT
Rpa2	Forward Primer	GAGTCCGAGCCCAGCATATTG
	Reverse Primer	CCTGTGAAATCTCGACATCTCCA
Xrcc5	Forward Primer	ATGGCGTGGTCCGGTAATAAG
	Reverse Primer	CCTGTCGTTGGACAAACATAGTC
Xrcc6	Forward Primer	ATGTCAGAGTGGGAGTCCTAC
	Reverse Primer	TCGCTGCTTATGATCTTACTGGT
53bp1	Forward Primer	GGGGAGCAGATGGACCCTA
	Reverse Primer	GGAAGGTGTCGAGATAGCACG
Prkdc	Forward Primer	AAACCTGTTCCGAGCTTTTCTG
	Reverse Primer	TAAGGGCACAGCATATCGCTT
Lig4	Forward Primer	ATGGCTTCCTCACAAACTTCAC
	Reverse Primer	TTTCTGCACGGTCTTTACCTTT
Nheg1	Forward Primer	TGGGCATGGTTACAACTTGC
	Reverse Primer	AACCGTGCTTGGTGATAGACA
GAPDH	Forward Primer	CCTCGTCCCGTAGACAAAATG
	Reverse Primer	TCTCCACTTTGCCACTGCAA
